# Functional Role of γ-Crystallin N in the Auditory Hindbrain

**DOI:** 10.1371/journal.pone.0161140

**Published:** 2016-08-12

**Authors:** Heiner Hartwich, Elena Rosengauer, Lukas Rüttiger, Viviane Wilms, Sarah-Kristin Waterholter, Hans Gerd Nothwang

**Affiliations:** 1 Neurogenetics group, Center of Excellence Hearing4All, School of Medicine and Health Sciences, Carl von Ossietzky University Oldenburg, 26111, Oldenburg, Germany; 2 University of Tübingen, Department of Otolaryngology, Hearing Research Centre Tübingen (THRC), Molecular Physiology of Hearing, Elfriede Aulhorn Str. 5, 72076, Tübingen, Germany; 3 Research Center for Neurosensory Science, Carl von Ossietzky University Oldenburg, 26111, Oldenburg, Germany; Institut de la vision, FRANCE

## Abstract

γ-crystallins are major components of the vertebrate lens but show expression in other tissues as well. Their extralenticular functions remain so far unclear. Here, we explored such roles in the rodent superior olivary complex in which previous analysis demonstrated developmentally regulated expression of *Crygd*, *Cryge* and *Crygn*. Immunohistochemistry with novel antibodies against Crygd/e and Crygn indicate that expression of *Crygd/*e was moderate and varied between the perinatal superior olivary complex of mice, rats, and gerbils. Crygn-immunoreactivity was more robust and consistently highest in the medial nucleus of the trapezoid body, but also present in other nuclei of the superior olivary complex. To analyze the function of Crygn in the auditory hindbrain, we used a *Crygn* allele with a floxed exon 2. Upon pairing with *Egr2*::*Cre* mice, exon 2, encoding the first two greek key motifs of Crygn, was deleted in the developing auditory hindbrain. Anatomical analysis of these mice revealed a 20% volume reduction in the medial nucleus of the trapezoid body and a 7% reduction in the lateral superior olive at postnatal day 25. This was due to cell loss between postnatal days 4 and 25, whereas cell size was unaffected. Auditory brainstem responses showed normal threshold but a significant increase in the amplitude of wave IV. *Crygn* is hence required for postmigratory survival and proper function of auditory hindbrain neurons. These results ascertain for the first time an essential extralenticular role for γ-crystallins *in vivo*.

## Introduction

γ-crystallins are small intracellular proteins of 174 to 182 amino acids with a molecular mass of ~21 kDa [1,2]. In vertebrates, four different classes of γ-crystallins exist: *Cryga-f*, *Crygm*, *Crygn*, *and Crygs* [1,2]. All family members share a highly symmetrical structure built from four characteristic Greek key motifs arranged into two similar domains [3]. Each Greek key motif consists of around 40 amino acids that fold into four anti-parallel β-strands [3]. Together with β-crystallins, γ-crystallins form the ancient βγ superfamily of crystallins, which is related to microbial spore coat proteins. In vertebrates, they account for the majority of the soluble proteins in lens [4,5]. Accordingly, γ-crystallins play a key role in determining the optical properties of this structure and mutations in several family members are associated with cataracts (*Crygb-e*, *Crygs*) or opacity (*Crygf*) in both humans and mice [2,6].

Outside the lens, γ-crystallins are expressed in the retina and in the nervous system such as the hippocampus [7], yet little is known concerning their function in these tissues. Large variations in expression levels between mouse strains and between eyes in the same animal as well as poor correlation of RNA and protein levels [7,8] even put in question the functional importance of non-lens expression [9]. We recently observed high perinatal expression of *Crygd*, *Cryge*, and *Crygn* in the rat superior olivary complex (SOC) [10]. The SOC is an important second-order auditory center in the hindbrain, involved in sound localization and processing of temporal information [11–14]. Of note, the three *γ-crystallin* genes showed down-regulation during postnatal development and had higher expression in the perinatal SOC compared to the age-matched total brain [10], pointing to an important role during development. This makes the SOC a promising system to study the role of γ-crystallins outside vision. We therefore set out to characterize in detail the expression pattern of γ-crystallins in the SOC on the protein level in three different rodents, using newly generated antibodies. To gain insight into the function of γ-crystallins, we also generated and analyzed a region-specific *Crygn* knockout mouse. Together, the data revealed that *Crygn* is required for integrity and function of the auditory brainstem. This describes for the first time an essential extralenticular role for γ-crystallins.

## Results

### Generation and validation of γ-crystallin antibodies

**Fig 1:**
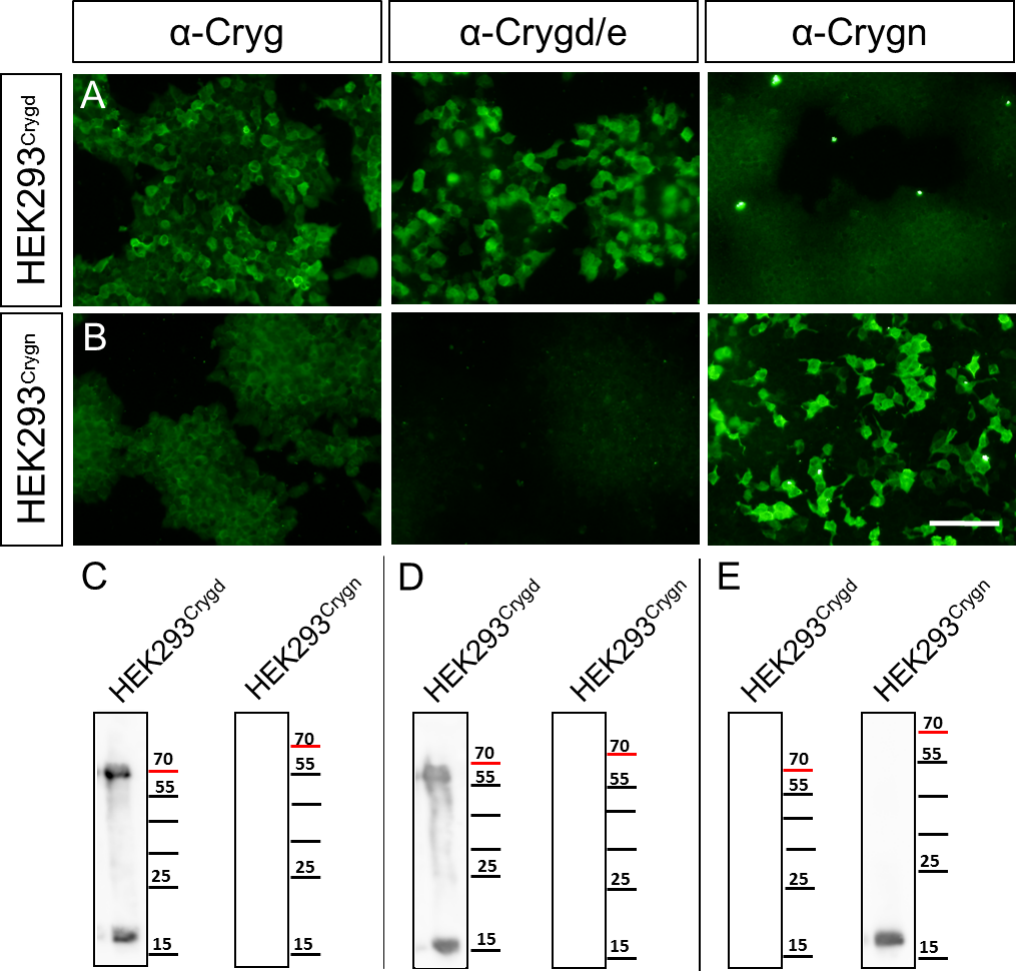
Validation of γ-crystallin antibodies in HEK293 cells. HEK293 transiently transfected with either *Crygd-flag* (HEK293^Crygd^) (**A**) or *Crygn-flag* (HEK293^Crygn^) (**B**) expression constructs were labeled with antibodies against Cryg (α-Cryg), Crygd/e (α-Crygd/e), or Crygn (α-Crygn). α-Cryg and α-Crygd/e both detect Crygd, but not Crygn in immunocytochemistry, whereas α-Crygn detects only Crygn. (**C-E**) Similar results were obtained in immunoblot analysis where α-Cryg and α-Crygd/e both detect Crygd, whereas α-Crygn detects Crygn. Scale bar is 100 μm.

To gain insight into the role of γ-crystallins in the developing SOC, we wished to perform immunohistochemistry. This approach is hampered by the lack of isoform-specific antibodies. We therefore generated two different antibodies: one antibody against Crygd and Cryge (anti-Crygd/e, no immunogenic peptide is specific to Cryge) and one specifically recognizing Crygn (anti-Crygn). To validate the antibodies, the open reading frames of *Crygd* or *Crygn* were cloned into expression vectors, which were transiently transfected into HEK293 cells. Subsequent immunocytochemistry revealed that anti-Cryg, previously been used in our analysis of the SOC [10] strongly recognized Crygd (Fig 1A). In contrast, the immunoreactivity against Crygn was close to background (Fig 1A). In the immunoblot analysis, the antibody only bound to Crygd, detecting both a 17 kDa and a 55 kDa band (Fig 1C). As none of the two signals was present in *Crygn* transfected cells, they represent monomeric and likely complexed Crygd. Anti-Crygd/e recognized Crygd and not Crygn (Fig 1A and 1B), whereas anti-Crygn recognized Crygn and not Crygd (Fig 1A and 1B). These specificities were also observed in immunoblot analyses. Anti-Crygd/e detected a band of approximately 16 kDa, corresponding to the monomer, and an additional band around 55 kDa only in *Crygd* transfected cells (Fig 1D), similar to the antibody anti-Cryg. Anti-Crygn detected a 16 kDa band, corresponding to the monomer, only after transfection of HEK293 cells with a *Crygn* expression clone (Fig 1E). These data reveal that the two novel antibodies allow distinction between Crygd/e and Crygn.

### Different immunoreactivity patterns of γ-crystallins in the perinatal rat, mouse, and gerbil SOC

**Fig 2:**
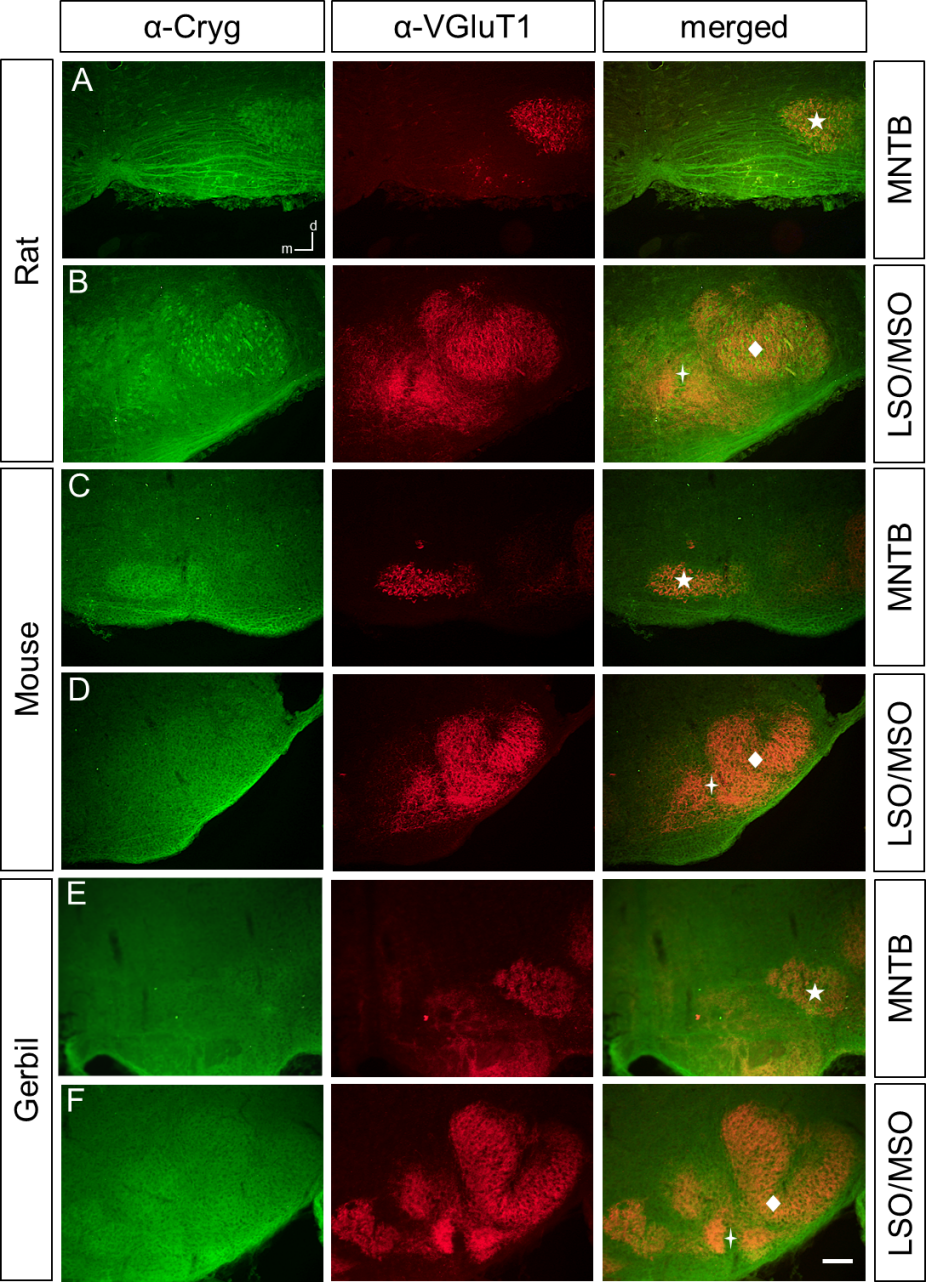
Cryg-immunreactivity in the rat, mouse, and gerbil SOC at P4. All image series show Cryg-ir (α-Cryg) in the first, VGluT1-ir (α-VGluT1) in the second, and an overlay of both immunoreactivities in the third column. This order applies also to subsequent Figs 3 to 5. (**A)** Cryg-ir is clearly seen in the MNTB and in the ventral acoustic stria of rat. (**B)** The MSO and a subpopulation of the LSO show also prominent Cryg-ir. (**C,D)** The mouse displays a weaker labeling in the SOC. (**E-F)** MNTB, LSO and MSO of the gerbil show no Cryg-ir above background. MNTB, medial nucleus of the trapezoid body; MSO, medial superior olive; LSO; lateral superior olive. MNTB, medial nucleus of the trapezoid body, designated by a asterisk MSO, medial superior olive, designated by a star; () LSO; lateral superior olive, designated by a diamond. The abbreviations and symbols also apply to subsequent figures. Dorsal is up and medial to the left. n = 3, scale bar is 100 μm.

**Fig 3:**
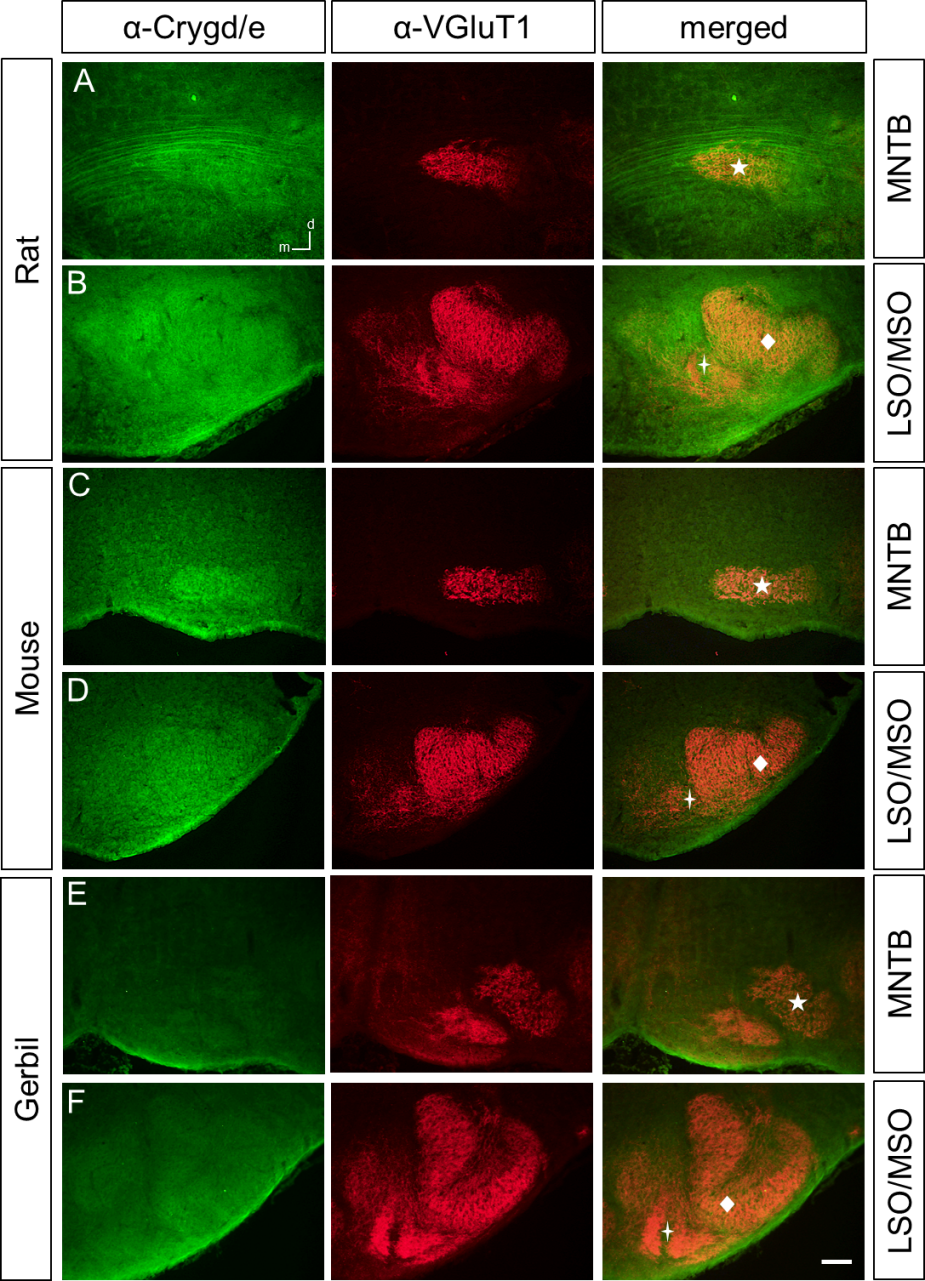
Crygd/e-ir in the rat, mouse, and gerbil SOC at P4. **(A)** Crygd/e labeling is observed in the rat MNTB and fibers of the acoustic stria. (**B)** LSO and MSO display moderate Cryge-ir. In the mouse, MNTB (**C**), LSO and MSO (**D**) are also labeled. (**E-F)** The gerbil MNTB, LSO and MSO show Crygd/e-ir similar to background. Dorsal is up and medial to the left. n = 3, scale bar is 100 μm.

**Fig 4:**
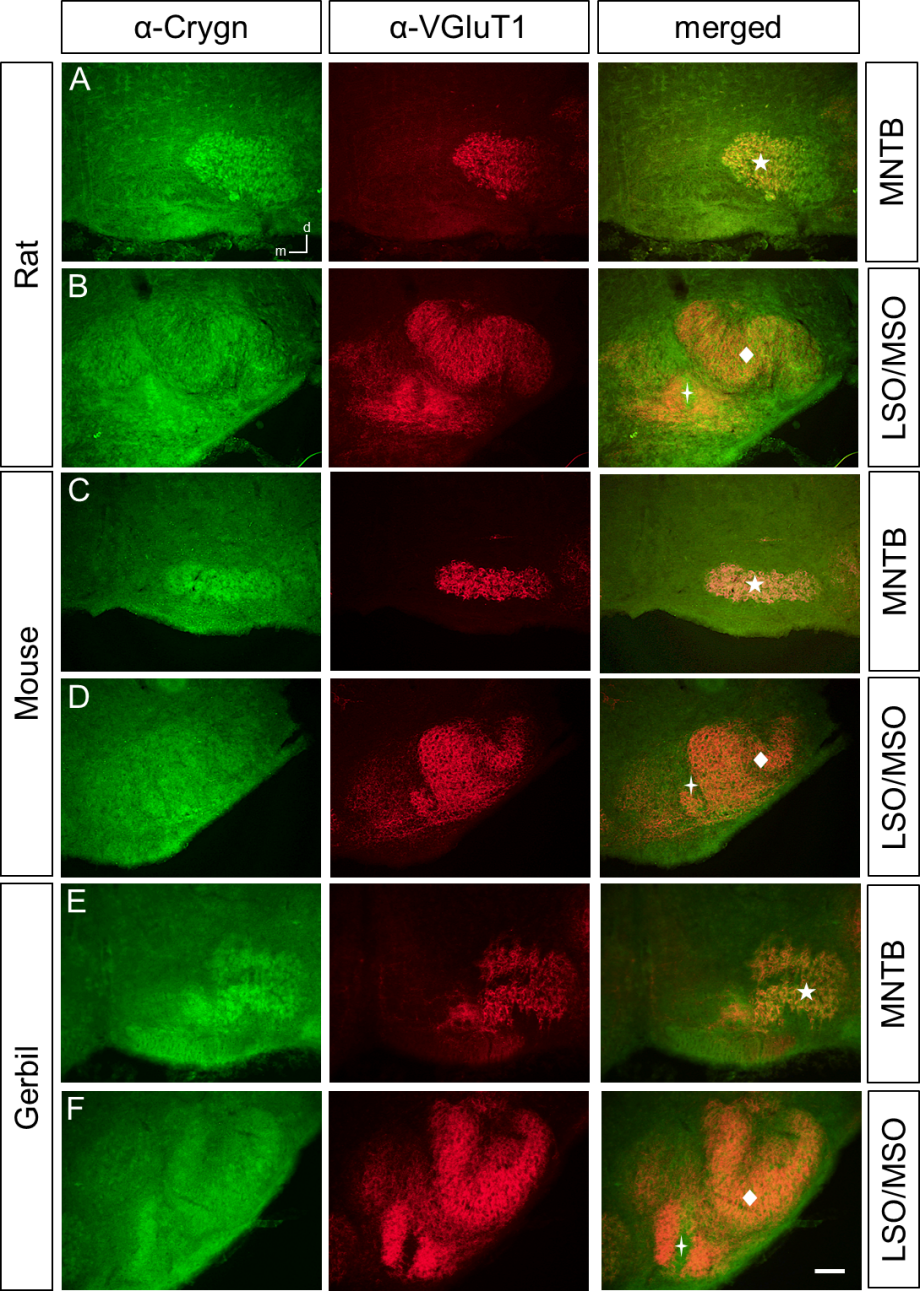
Crygn-ir in the rat, mouse, and gerbil SOC at P4. Crygn clearly labels the MNTB (**A**), the LSO and the MSO (**B**) of rat. (**C)** The mouse MNTB shows a moderate labeling and the LSO and MSO a weak immunoreactivity (**D**). (**E)** Gerbils show a similar pattern of Crygn-ir as the mouse with the MNTB being strongest labeled. Dorsal is up and medial to the left. n = 3, scale bar is 100 μm.

We next employed the three antibodies to study the expression of γ-crystallins in the rat and mouse perinatal SOC at P4. To clearly detect SOC structures, we co-labeled with an antibody against the vesicular glutamate transporter VGluT1, a presynaptic marker for SOC nuclei [15–17]. We focused our analysis on the lateral and medial superior olive (LSO and MSO), respectively, and the medial nucleus of the trapezoid body (MNTB), as they are major nuclei of sound localization pathways. In the rat, anti-Cryg labeled prominently the MNTB, the fibers of the ventral acoustic stria, the MSO, and a large population of neurons in the LSO (Fig 2A and 2B). In the mouse, immunoreactivity was clearly seen in the MNTB and weak in the LSO, MSO, and the acoustic stria (Fig 2C and 2D). For the newly generated anti-Crygd/e antibody, labeling was observed throughout the SOC in the rat and mouse. Similar to anti-Cryg, fibers of the ventral acoustic stria were labeled in the rat but not in the mouse (Fig 3A–3D). Finally, anti-Crygn clearly labeled the rat MNTB, MSO, and neurons of the LSO (Fig 4A and 4B). In the mouse, MNTB showed a moderate immunoreactivity and the LSO and MSO were weakly labeled (Fig 4C and 4D).

**Fig 5:**
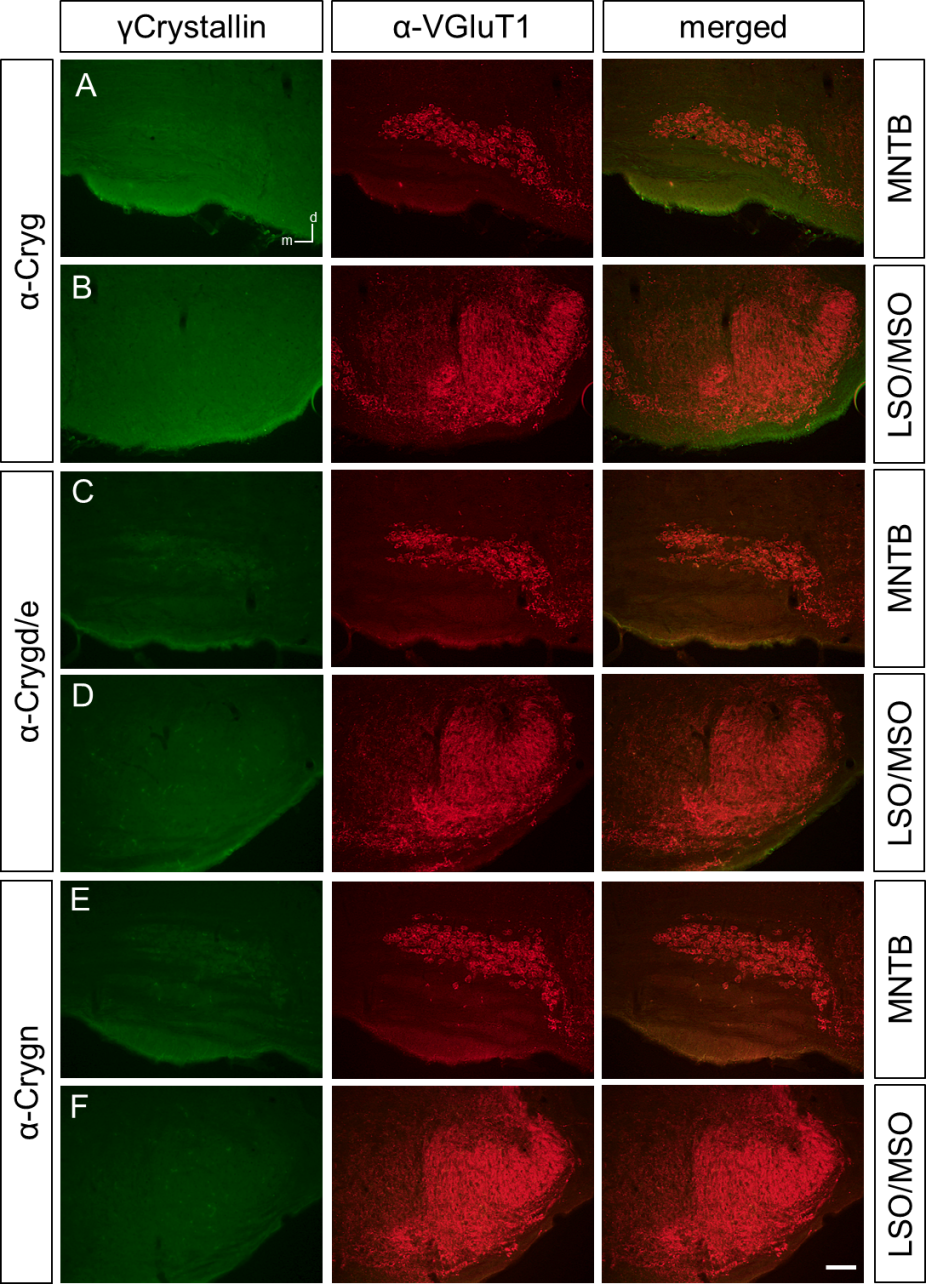
Cryg-ir in the mouse SOC at P25. All three antibodies against crystallins, i.e. Cryg (**A,B**), Crygd/e (**C,D**) or Crygn (**E,F**) gave no signals above background in the MNTB, the LSO and the MSO of mice aged P25. Dorsal is up and medial to the left. n = 3, scale bar is 100 μm.

In general, immunoreactivity for all three antibodies was weaker in the mouse compared to the rat. We therefore included the gerbil as a third rodent to obtain a broader view on γ-crystallin expression in rodents. For both anti-Cryg and anti Crygd/e, immunoreactivity was even weaker than in the mouse (Figs 2E, 2F, 3E and 3F). Anti-Crygn immunoreactivity was strongest in the gerbil MNTB (Fig 4E), but also present in the MSO and LSO (Fig 4F). One explanation for the weaker expression of γ-crystallins in mice compared to rats might be differently timed developmental processes [18]. To explore this possibility, we also immunolabeled prenatal mice between embryonic days 15 to 18. This analysis yielded only immunoreactivity close to background (data not shown). Finally, we investigated the expression of the different γ-crystallins in the adult SOC by applying the three antibodies to sections of P25 old mice. No significant labeling was obtained (Fig 5), confirming postnatal down-regulation of *γ-crystallins* in the SOC [10].

### Generation and validation of a *Crygn*^*Egr2*^ mouse line with deletion of exon 2

**Fig 6:**
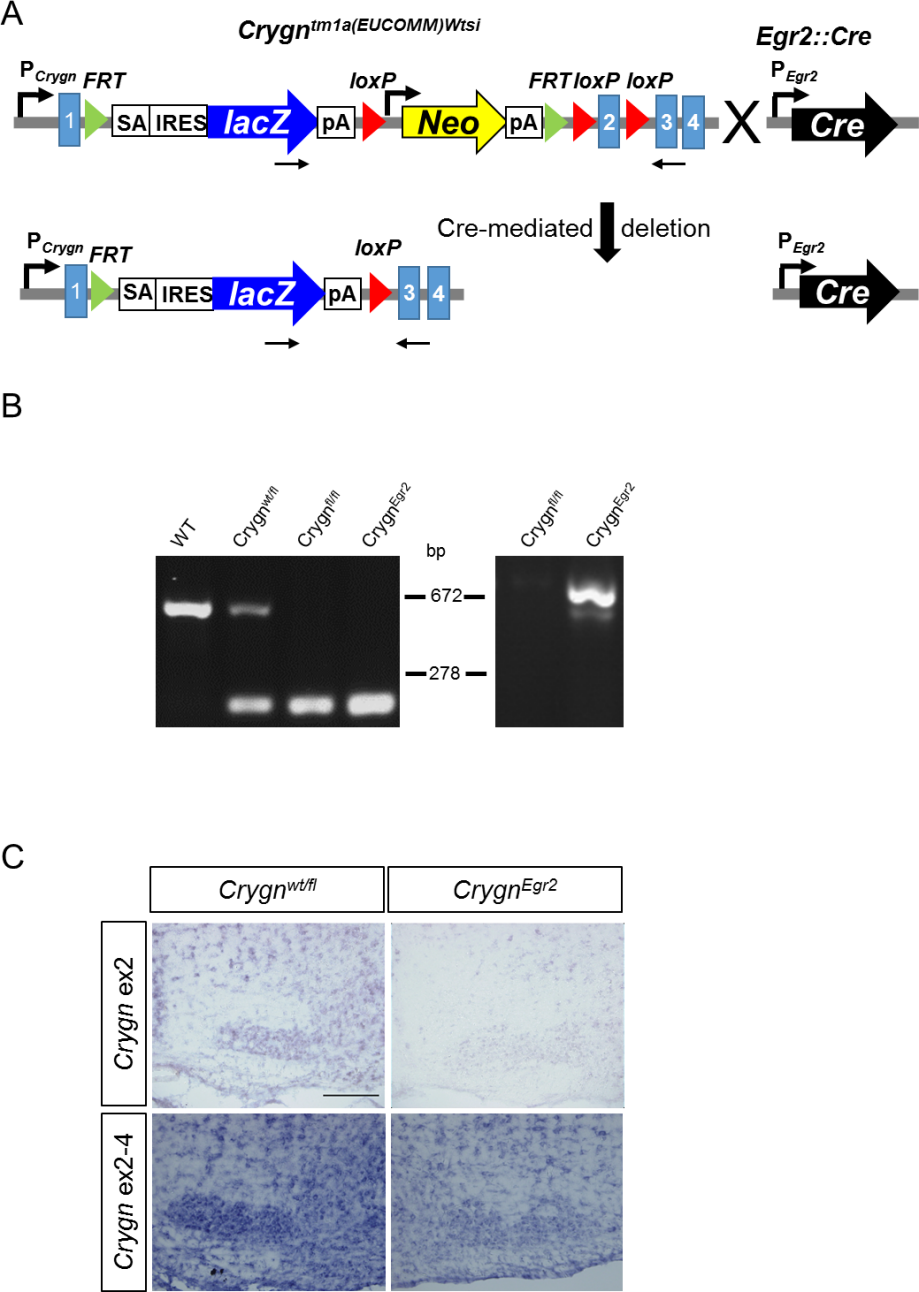
Generation of a spatially restricted *Crygn* knockout mouse in the auditory hindbrain. **(A)** Scheme of the knockout strategy, consisting of crossing a mouse line with a floxed allele of exon 2 of *Crygn* (*Crygn*:*tm1a* (EUCOMM)) and the *Egr2*::*Cre* driver line. Primers for probing recombination are depicted as black arrows. (**B)** Validation of the spatial ablation in the SOC. Left side: Genotyping of the floxed *Crygn* locus. In wt, a 524 bp long PCR product is amplified, whereas the mutant locus results in a 207 bp product. Right site: Confirmation of recombination in the SOC. Upon recombination, a 604 bp long product is amplified. The non-recombined locus is 3,411 bp in length and not amplified under the PCR conditions used. (**C)** RNA *in situ hybridization* analysis in the MNTB. An RNA probe complementary to exon 2 yields only signals in MNTB section of control mice whereas no signal is observed in *Crygn*^*Egr2*^ mice. In contrast, an RNA probe complementary to exons 2–4 still yields signals in the MNTB of *Crygn*^*Egr2*^ mice. This indicates transcription of the truncated *Crygn* gene. Scale bar is 200 μm.

Our analyses so far revealed, that anti-Crygn clearly labeled the SOC in all three rodents at P4, whereas the other two antibodies mainly directed against Crygd/e gave only modest immunoreactivity in mouse and gerbil. Furthermore, *Crygd* and *Cryge* are pseudogenes in humans [6], which renders the extrapolation of functional data obtained in mice to other mammals difficult. We therefore focused our functional analysis on *Crygn* using a transgenic mouse line with a floxed *Crygn* allele generated by the International Knockout Mouse Consortium [19] (Fig 6A). The modified *Crygn* locus harbors a *lacZ* gene and a *Neo* cassette after exon 1 and *loxP* sites flanking exon 2 (Fig 6A). This allele design represents a so-called ‘‘knockout-first” allele due to the insertion of *LacZ* and the *Neo* cassette, which interrupts transcription of *Crygn* [19]. This cassette design should result in expression of *lacZ*, encoding β-galactosidase under the *Crygn* promoter. To corroborate our immunohistochemical data, we wished to exploit this reporter activity. Yet, X-gal (5-bromo-4-chloro-3-indolyl-b-D-galactopyranoside) staining failed to detect β-galactosidase activity throughout the entire brain (data not shown). Preliminary PCR analyses indicate that part of the 5’ end of the *LacZ* gene has been lost (data not shown). As also *Crygn* mRNA could be detected in the brain of homozygous *Crygn*^*fl/fl*^ mice (data not shown and Fig 6C), indicating a corrupted 5’ end of the inserted construct, we decided to employ a conditional knockout strategy by crossing the floxed *Crygn*^*fl*^ allele with the *Egr2*::*Cre* driver line, which recombines in rhombomeres 3 and 5 derived neurons [20] (Fig 6A). In this cross, named *Crygn*^*Egr2*^, exon 2, encoding the first two greek key motifs, should be absent in most parts of the SOC, the anterior ventral cochlear nucleus, harboring the major input neurons into the SOC, and the dorsal cochlear nucleus, projecting to the inferior colliculus [17,21–25].

To confirm recombination, a PCR strategy was designed which amplifies a 604 bp long product upon recombination, whereas the non-recombined locus with 3,411 bp in length will not be amplified under the PCR conditions used. We used this strategy to probe genomic DNA isolated from the SOC of control or *Crygn*^*Egr2*^ mice. A 604 bp PCR product was clearly present when using genomic DNA from the SOC of *Crygn*^*Egr2*^ mice, whereas no product was obtained for SOC DNA from control mice (Fig 6B). The absence of exon 2 was confirmed by RNA *in situ* hybridization. A probe complementary only to the sequence of exon 2 hybridized to sections derived from control mice but yielded no signals from corresponding sections of *Crygn*^*Egr2*^ mice (Fig 6C). In contrast, a probe complementary to exons 2–4 hybridized to the SOC from *Crygn*^*Egr2*^ mice, albeit yielding weaker signals than in heterozygous *Crygn*^*wt/fl*^ control mice (Fig 6C). These results reveal that exons 1, 3, and 4 are still transcribed from the modified locus and that the original allele in *Crygn*^*fl*^ mice represents thus not a knockout-first allele. This is in agreement with the observation of *Crygn* mRNA in homozygous *Crygn*^*fl/fl*^ mice and the lack of functional β-galactosidase.

Together, these data demonstrate that *Crygn*^*Egr2*^ mice lack exon 2 in the auditory hindbrain, that encodes the first two greek motifs in domain 1 except for the first two amino acids [1]. However, we note that lack of exon 2 does not disrupt the open reading frame, as it represents a phase 0–0 symmetric exon [26]. Due to transcription of the truncated *Crygn* mRNA, there is some residual truncated Crygn protein present within the auditory hindbrain. This is supported by the fact that our Crygn antibody labeled the SOC in *Crygn*^*Egr2*^ mice (data not shown). This prevented testing the specificity of the Crygn antibody in this mouse model.

### Structural abnormalities in the adult SOC of *Crygn*^*Egr2*^ mice

**Fig 7:**
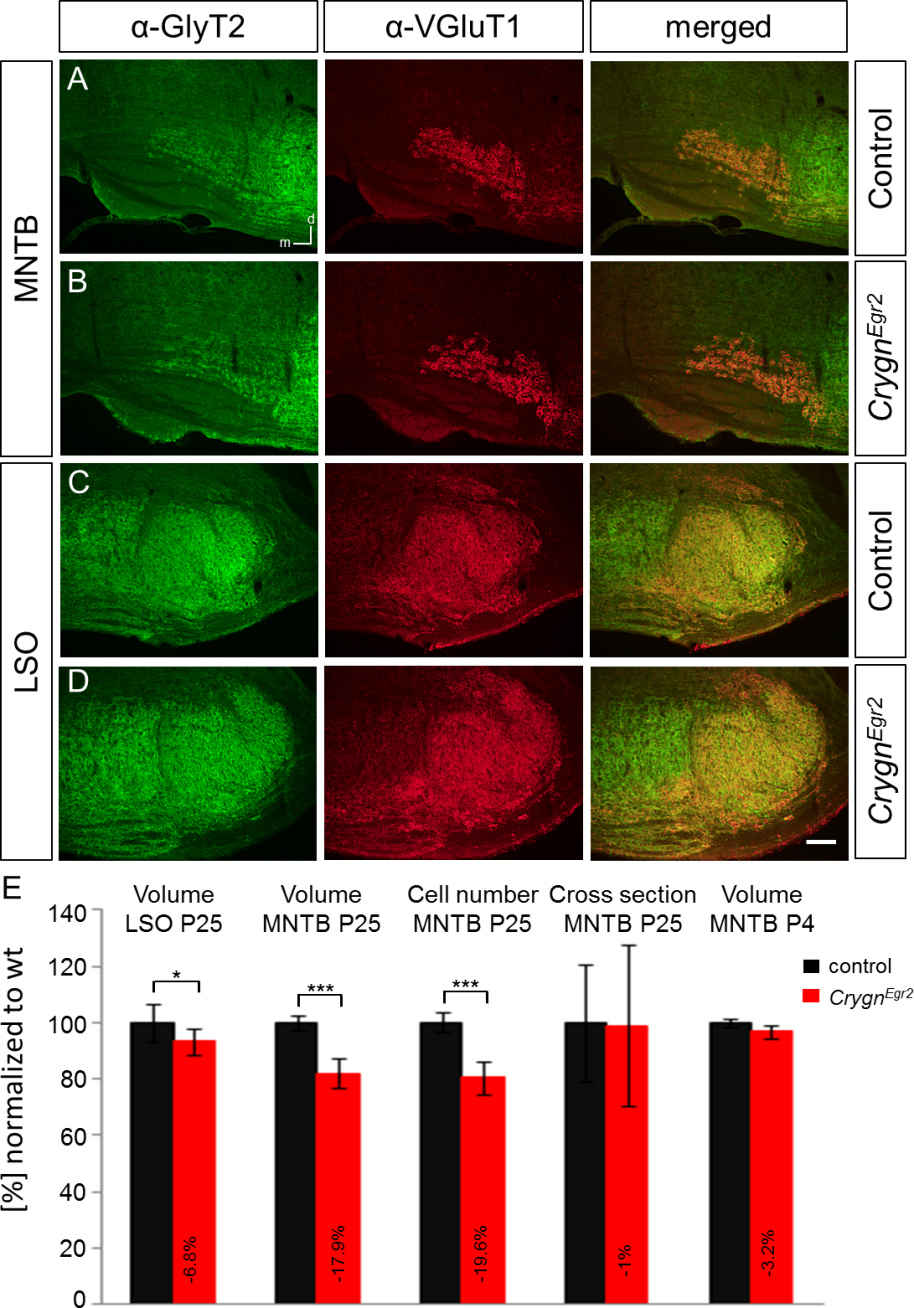
Immunohistochemical and anatomical analysis of the SOC in *Crygn*^*Egr2*^ mice. (**A**) Normal gross anatomy of the SOC in *Crygn*^*Egr2*^ mice. GlyT2 and VGluT1 immunoreactivity in coronal brainstem sections of P25 *Crygn*^*Egr2*^ mice and control litter mates indicate normal gross morphology of SOC nuclei. (**B**) Structural alterations in nuclei of the SOC. In *Crygn*^*Egr2*^ mice, the volumes of the LSO and MNTB were significantly decreased. The MNTB of *Crygn*^*Egr2*^ mice displayed also lower cells number but normal cross sectional area of neurons. At P4, MNTB cell number was not affected by the genotype. Volume and cell number were analysed in Nissl-stained serial sections of the respective nucleus (6 nuclei from 3 animals/genotype). As a test for statistical significance, a two-tailed student’s *t*-test was used. Color coding (black: control control litter mice; red: *Crygn*^*Egr2*^ mice). Dorsal is up and medial to the left. * *p* ≤ 0.01; ***p ≤ 0.001.

To probe the functional consequences resulting from the loss of *Crygn* exon 2, we first investigated the integrity of the SOC in young-adult P25 mice by fluorescent immunohistochemistry for VGluT1 and the glycine transporter GlyT2, a presynaptic marker for inhibitory synapses in the auditory system [27–30]. Immunohistochemical analysis of the SOC using these two antibodies revealed no difference between *Crygn*^*Egr2*^ and control littermates containing one wt-allele and the *Egr2*::*Cre* allele (Fig 7A–7D). All major nuclei of the SOC were clearly labeled and no gross abnormalities in size or shape were observed. However, quantitative analysis of Nissl-stained sections demonstrated a significant volume reduction of the MNTB by 19.6% (control: 0.0392 ± 0.0013 mm^3^; *Crygn*^*Egr2*^: 0.0315 ± 0.0019 mm^3^, *p* = 0.0000094), whereas the LSO showed a small but significant reduction of 7% (control: 0.0444 ± 0.0025 mm^3^; *Crygn*^*Egr2*^: 0.0413 ± 0.0016 mm^3^, *p* = 0.0285) (Fig 7E).

Reduced volume could be the consequence of decreased cell number, reduced cell size, or changes in cell density. To distinguish between these possibilities we focused on the MNTB, showing the strongest phenotype. Cell counts revealed that the decreased volume in the MNTB was due to a 19.6% reduction in the number of neurons (control: 3,085.8 ± 108.3; *Crygn*^*Egr2*^: 2,481.8 ± 182.8, *p* = 0.00004) (Fig 7E). In contrast, analysis of cross sectional area of MNTB soma revealed no reduction (control: 153.31 ± 31.71 μm^2^; *Crygn*^*Egr2*^: 151.73 ± 44.17 μm^2^, *p* = 0.7136) (Fig 7E).

The observed reduction in cell number in the MNTB can be due to defects in cell birth, migration, or postmigratory survival of neurons. To assess which of these steps requires Crygn, we analyzed the MNTB at P4, five to six days after completion of migration [31]. This nucleus showed the strongest volume reduction and can easily be delineated at perinatal stages in the mouse [31]. Quantitative analysis of Nissl-stained sections revealed normal volume in *Crygn*^*Egr2*^ mice (control: 0.0216 ± 0.0003 mm^3^; *Crygn*^*Egr2*^: 0.0209 ± 0.0005 mm^3^, *p* = 0.0615) (Fig 7E). Together, these data demonstrate that Crygn is required for postnatal integrity of second-order auditory hindbrain neurons. Furthermore, the postmigratory effect observed in *Crygn*^*Egr2*^ mice argue against a contribution of *Egr2* haploinsufficiency in the *Egr2*::*Cre* driver line to the observed phenotype. This is in agreement with the lack of anatomical abnormalities in heterozygous *Egr2*::*Cre;Cacna1d*^*wt/fl*^ mice [22].

### Altered auditory brainstem responses in *Crygn*^*Egr2*^ animals

**Fig 8:**
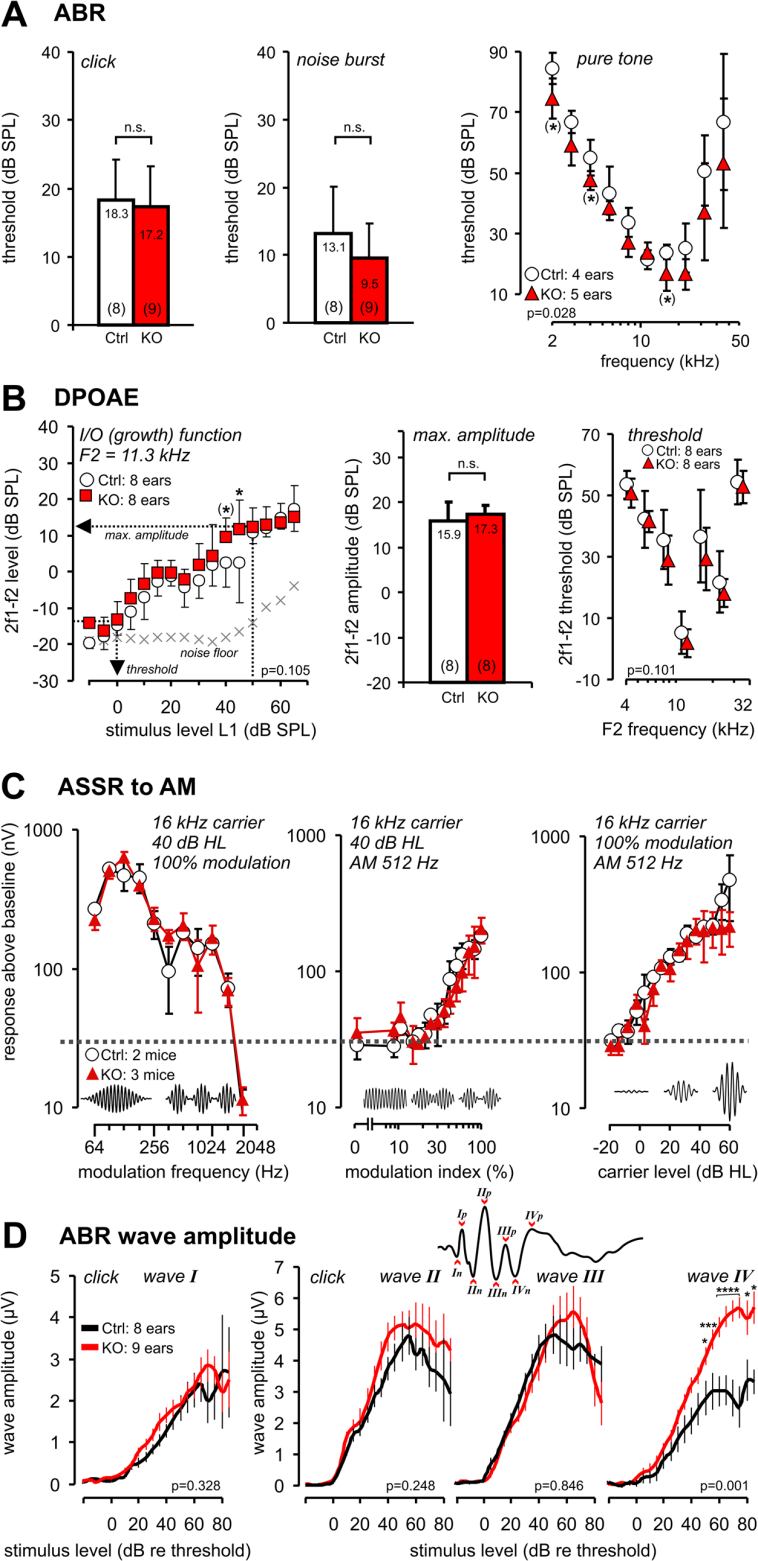
Altered auditory brainstem responses (ABR) in *Crygn*^*Egr2*^ mice. (**A**) Auditory brainstem response (ABR) thresholds for click, noise burst, and pure tone stimuli for control (white) and *Crygn*^*Egr2*^ mice (red) (n = 4 and 5 mice for controls and *Crygn*^*Egr2*^, respectively). No difference was observed for ABR thresholds in response to click stimuli (left) or noise burst stimuli (middle panel, n.s., not significant, p > 0.05). Threshold for pure tone stimuli were slightly but significantly better in *Crygn*^*Egr2*^ mice (*p* = 0.0279, 2-way ANOVA comparing the genotype). (**B**) Outer hair cell function measured by distortion product otoacoustic emission (DPOAE) growth function (left), maximal signal strength (middle, max. amplitude) and threshold for the 2f1-f2 distortion product (right). *Crygn*^*Egr2*^ mice had slightly improved 2f1-f2 distortion products indicated by increased amplitudes at 45 dB SPL stimulation (left, *p* = 0.1048, 2-way ANOVA) and small though non-significant improvements of DPOAE thresholds (right, *p* = 0.1013, 2-way ANOVA). This indicated intact outer hair cells and cochlear amplification in both genotypes. Arrows in left panel illustrate how threshold and amplitude were determined from the individual growth functions. (**C**) Processing of fast temporal modulation was measured by auditory steady state responses (ASSR) to amplitude modulated stimuli of increasing modulation speed (left, modulation frequency), as function of the modulation index (middle, modulation depth in %,) and for increasing level of the carrier (right, -20 to 60 dB hearing level, HL). For both genotypes, responses dropped for stimulation speeds above 1,024 Hz and were lost for faster modulation (2,048 Hz). Detection thresholds for modulated stimuli were at ca. 3–4% modulation, and response strength increased with carrier level. There was a tendency for *Crygn*^*Egr2*^ signals to level off at lower signal strength than the control (right panel, 55–60 dB hearing level), though this was not statistically significant. Insets schematically illustrate the used stimuli. (**D**) Changes of average peak amplitude for ABR wave I to IV (defined as peak to peak amplitudes, illustrated in the inset showing an example of an ABR recording with marked negative (n) and positive (p) peaks of either wave. The growth function shows significantly increased ABR amplitudes at wave IV at higher stimulus levels (> 50 dB, *p* < 0.001, 2-way ANOVA). Growth functions for ABR wave I and II amplitudes also were significantly changed, corroborating the results from the slightly improved ABR thresholds and DPOAE functions in *Crygn*^*Egr2*^ mice. Data for controls are shown as open bars, symbols and black lines, data for *Crygn*^*Egr2*^ mice are shown in red. Data represent mean and standard deviation (A,B) or standard error of the mean (C,D). The number of measured ears is indicated in each panel. n.s., not significant, *: p < 0.05, ***: p < 0.001, ****: p < 0.0001 in Holm-Sidak's multiple comparisons test. (*) indicates statistical results from uncorrected single comparison with *p* < 0.05 in 2-sided t-test.

To assess the functional consequences of the loss of *Crygn* exon 2 and the reduction in cell numbers of distinct SOC nuclei for auditory information processing, we audiometrically analyzed the hearing function of *Crygn*^*Egr2*^ and control mice from their auditory brainstem responses (ABR), otoacoustic emissions (DPOAE), and auditory steady state responses (ASSR). From ABR to click, noise burst, and pure tone stimuli, hearing thresholds (Fig 8A) and amplitude and latency growth functions for individual waves (Fig 8D) were determined. Thresholds for click and noise burst stimuli were similar in both genotypes and did not significantly differ (*p* = 0.16 and *p* = 0.09 for click and noise stimuli, respectively, 2-sided t-test). There was a tendency for even slightly better thresholds upon pure tone stimuli in the *Crygn*^*Egr2*^ mice (*p* = 0.0279 for factor genotype, F(1, 7) = 7.64, 2-way ANOVA). However, differences were small (ca. 8 dB on average, refers to an effect size of D = 1.5) and post-tests did not disclose a particular stimulus frequency at which thresholds were different (p > 0.05, Holm-Sidak's multiple comparisons test). As expected from the ABR thresholds, outer hair cells function as determined by DPOAE growth functions, amplitudes, and thresholds (Fig 8B) also were slightly though non-significantly better in *Crygn*^*Egr2*^ mice than in controls (growth function at f2 = 11.3 kHz: *p* = 0.1048 for factor genotype, F(1, 15) = 2.98, 2-way ANOVA). Pairwise comparison revealed larger responses in *Crygn*^*Egr2*^ mice than in controls only at 45 dB SPL (*p* < 0.05, Holm-Sidak's multiple comparisons test). The maximal emission strength was not significantly different (50 dB SPL L2: *p* = 0.4, 2-sided t-test). Corresponding to the slightly improved ABR thresholds in *Crygn*^*Egr2*^ mice, DPOAE thresholds were slightly but non-significantly improved in *Crygn*^*Egr2*^ mice (*p* = 0.1013 for factor genotype, F(1, 14) = 3.08, 2-way ANOVA), proposing an increased outer hair cell response as the origin of the slightly better ABR (hearing) thresholds in *Crygn*^*Egr2*^ mice. This slightly improved responsiveness of the sensory structures in the cochlea may be due to a reduced activity within the inhibitory olivo-cochlear efferent feedback upon outer hair cells and inner hair cell afferents, resulting in marginally better thresholds, and stronger outer hair cell responses at close threshold and moderate stimulation levels (45 dB SPL).

Since temporal processing depends on the fast and synchronous responses of neurons in hindbrain and midbrain auditory structures, the observed anatomical alterations in *Crygn*^*Egr2*^ mice might impair temporal auditory processing in these mice. Therefore the temporal responses were recorded in *Crygn*^*Egr2*^ and control mice by auditory steady state responses (ASSR) to amplitude modulated stimuli (AM) and by calculating the amplitudes and latencies of subsequent waves in the auditory brainstem response (waves I, II, III, and IV) that are generated by synchronous neuronal activation within the hierarchically organized succeeding auditory brainstem nuclei (wave I: auditory nerve, wave II: cochlear nucleus complex, wave III: SOC, wave IV: lateral lemniscus and inferior colliculus [32,33]). ASSR for amplitude modulated stimuli of increasing modulation frequency (64–2,028 Hz), increasing modulation depth (index 1% - 100%), and increasing carrier stimulus level did not reveal any deficits in the capacity of *Crygn*^*Egr2*^ mice to process temporally modulated auditory stimuli (Fig 8C). Best responses in the temporal modulation transfer function occurred within the same modulation frequencies as for control mice (ca. 128 Hz) and responses brake off at similar high modulation frequencies (> 1,024 Hz). Modulation thresholds were not different for both groups (ca. 3–4% modulation index) and modulation responses extended to the same range in both genotypes (carrier levels up to 40 dB hearing level). There was a tendency for a signal compression in *Crygn*^*Egr2*^ mice at higher carrier levels while the response in control mice still grew (50–60 dB hearing level, Fig 7C, right panel). Corroborating the normal or slightly improved cochlear and cochlear nucleus function, waves I and II amplitudes of ABRs were normal or even slightly increased for *Crygn*^*Egr2*^ mice (Fig 8D, wave I: *p* = 0.3287 for factor genotype, F(1, 15) = 1.02, wave II: *p* = 0.2476 for factor genotype, F(1, 15) = 1.45, 2-way ANOVA, no significance for pair-wise comparison in post-test). Wave III amplitudes were not different for *Crygn*^*Egr2*^ and control mice (*p* = 0.8461 for factor genotype, F(1, 13) = 0.0392). In contrast, the amplitudes of wave IV, which reflects activity in the lateral lemniscus and inferior colliculus [32,33], were significantly increased above 50 dB in *Crygn*^*Egr2*^ mice compared to control littermates (Fig 8D, *p* < 0.001 for factor genotype, F(1, 11) = 19.6, 2-way ANOVA). Overall latencies of ABR peaks were not altered (data not shown). The increased wave IV amplitude indicates an altered input from the cochlear nucleus complex and/or SOC into higher order nuclei in *Crygn*^*Egr2*^ mice. Taken together these physiological data reveal that *Crygn*^*Egr2*^ mice display normal hearing sensitivity of the inner ear (as demonstrated by ABR thresholds and DPOAE functions), but that *Crygn* is essential for proper function in the auditory brainstem.

## Discussion

Here we report on the analysis of γ-crystallins in an extralenticular system. Using a region-specific knockout strategy, our data demonstrate that lack of exon 2 of *Crygn* in the auditory hindbrain causes reduction in volume and cell number of auditory nuclei as well as abnormal ABRs. These data reveal that Crygn plays an essential role for integrity and function of auditory nuclei. This establishes a requirement for γ-crystallins outside the lens.

### Crygn is required for postmigratory integrity of the SOC

Our anatomical analysis revealed volume reduction in both the MNTB (~20%) and LSO (7%) of *Crygn*^*Egr2*^ mice. A detailed analysis demonstrated that the volume reduction in the MNTB was due to cell death (Fig 7). This cell death occurs during terminal postnatal differentiation, after completion of migration [12]. Interestingly, Crygn is thus required for survival after the previously established period between P0 and P4, in which a significant number of SOC neurons die in the absence of innervation [23] or postsynaptic Ca^2+^ signaling [21,22]. Our data therefore extend the vulnerable period of second-order neurons of the auditory hindbrain to later postnatal stages. This is in agreement with the observation in mice lacking the neural cell adhesion molecule contactin 5 [34]. These mice showed cell death of MNTB neurons between P6 and 1 month of age [34]. This was likely due to a reduced number of calyces of Held, which represent the large presynaptic terminals of MNTB neurons [35,36]. A prolonged period of vulnerability beyond P4 was also shown for the anterior ventral cochlear nucleus, a first-order auditory nucleus in the hindbrain. Timed cochlear ablation experiments demonstrated an abrupt age-dependent change in sensitivity of these neurons to disruption of afferent input. Deafferentiation before P11 resulted in significant cell loss, whereas the same procedure done by age P14 had no effect on survival [37]. Thus, both first-order cochlear nucleus neurons and second-order SOC neurons display a period of vulnerability beyond the first days post-migration.

What might be the underlying mechanism of cell death in mice lacking Crygn? Recent analysis in mice lacking L-type Ca^2+^ channels revealed that MNTB neurons crucially depend on Ca^2+^ signaling. This was shown both *in vivo* by the use of mice lacking L-type Ca^2+^ channels [21,22,38] or *in vitro* by pharmacological approaches [39]. Due to the importance of Ca^2+^ signaling and homeostasis, developing auditory neurons express various Ca^2+^-binding proteins such as parvalbumin, calbindin, and calretinin [40,41]. Of note, in diverse microorganisms, members of the crystallin βγ-superfamily bind Ca^2+^ [42,43]. Yet, the Ca^2+^ binding site is degenerated in vertebrate family members, resulting in a low affinity Ca^2+^ binding site [44]. Consequently, no bound Ca^2+^ has ever been observed in crystal structures of lens βγ-crystallins [45]. Vertebrate βγ-crystallins likely traded the Ca^2+^ binding site for improved stability [44,45]. It is therefore unlikely that Crygn is critical for Ca^2+^ homeostasis in SOC neurons. This is in agreement with the different time periods of cell death in mice lacking Crygn (after P4) or L-type Ca^2+^ channels (P0—P4).

Alternatively, Crygn might have a presynaptic role. Β-crystallin family members were previously shown to be present in filopodial protrusions and axons of retinal ganglion cells and to promote axon regeneration [46–48]. Furthermore, β-crystallins were observed in neurites and growth cones of hippocampal neurons [46]. We did not observe Crygn in growth cones during prenatal development (data not shown) and only in the acoustic stria of rats, but not in other species. This renders such a presynaptic role questionable. The identification of the precise function of Crygn therefore requires further studies.

### Altered ABR in Crygn^Egr2^ mice

The analysis of auditory brainstem responses revealed a significant increase of the amplitude of wave IV, without detectable differences in the ABR thresholds or peak latencies (Fig 8). Wave IV likely reflects activity of the nuclei of the lateral lemniscus and the inferior colliculus [32,33]. Both nuclei are not targeted by the *Egr2*::*Cre* driver line [17], as their neurons are born outside r3 and r5 [49]. This suggests that the observed increase in amplitude reflects altered input from lower auditory centers. This assumption is compatible with the reduced number of MNTB neurons in *Crygn*^*Erg2*^ animals. MNTB neurons make inhibitory projections to various nuclei, including the LSO, the MSO, and the superior paraolivary nucleus within the SOC, and to the nuclei of the lateral lemniscus, which all project to the inferior colliculus [50–53]. Lack of inhibitory MNTB neurons therefore likely causes increased activity in the lateral lemniscus and inferior colliculus. However, this explanation cannot fully account for the phenotype, as indicated by anatomical and functional analyses in *contactin 5* null mice and *En1*^*Egr2*^ conditional knockout animals. *Contactin 5* null mice, which lack 10% of MNTB neurons, show mainly an increased interpeak latency between waves III and IV, whereas the amplitude of wave IV was unchanged [34]. In *En1*^*Egr2*^ mice, which lack the transcription factor En1 in r3 and r5 derived cells, the MNTB is entirely absent [54]. These animals exhibit a decreased wave III amplitude, whereas waves I, II, and IV were unaffected [54]. Thus cell loss in the MNTB alone cannot account for the altered amplitude of wave IV in our mouse model. It is thus likely, that molecular alterations contribute to the observed phenotype in *Crygn*^*Erg2*^ mice. This is in agreement with a comparative analysis of *Canca1c*^*Erg2*^ and *Cacna1d*^*Egr2*^ mice, which lack the L-type Ca^2+^ channels Ca_v_1.2 or Ca_v_1.3, respectively, in the auditory hindbrain. Despite a similar cell loss in major nuclei of the SOC such as 46% (*Canca1c*^*Erg*^) and 53% (*Cacna1d*^*Egr2*^) in the LSO, alterations in ABRs markedly differed between these two mouse lines. Loss of Ca_v_1.2 increased interindividual variation in latency of the negative peaks III and IV [21], whereas lack of Ca_v_1.3 resulted in increased amplitudes of waves II and III [22]. Altogether, these data support the notion that altered ABRs do often only poorly correlate with structural abnormalities in the auditory hindbrain.

### Species-specific expression pattern of γ-crystallins

Our immunohistochemical analyses revealed considerable differences in the immunoreactivity pattern and signal strength between rats, mice, and gerbils. This could reflect species-specific differences in the affinity of the antibodies used. However, crystallins are highly conserved and species-specific immunolabeling patterns were observed for all three antibodies (Figs 2–4). It is therefore more likely that our species-specific differences add to the reported highly variable expression of γ-crystallins between species or even strains. Considerable variability is observed between vertebrates, with fish and rodents showing high levels, whereas other terrestrial species show much lower levels [1]. Some γ-crystallins have even been converted to pseudogenes during evolution such as *Cryge and Crygf* in humans [1]. Finally, even mouse strain-specific differences exist with high levels of γ-crystallins in the retina of C57BL/6 mice and relatively low levels in DBA/2J animals [8]. It will therefore be interesting to study whether Crygn has a similar function in the auditory brainstem of other mammals such as the gerbil or the rat.

*γ-crystallins* expression in the retina was suggested to be regulated by Maf transcription factors, based on a bioinformatic analysis [7]. Subsequent studies in various Maf transcription factor knockout mice such as a *MafA/MafB* double knockout mouse line observed no alteration in the expression of γ-crystallins [55]. This points to a Maf-independent expression of γ-crystallins in the retina. Our data in the brainstem conform to this assumption. Expression of *γ-crystallins* is generally higher in the MNTB compared to the LSO and MSO, whereas expression of MafB is high in the latter two nuclei, but absent in the MNTB [56]. Thus, other transcription factors than MafB have to account for the different expression of *γ-crystallins* within the SOC and between rodents.

In summary, our data add *Crygn* to genes such as *Cacna1c* [21], *Cacna1d* [22], *contactin-5* [34] or *En1* [57,58], that are important for maintenance of auditory hindbrain structures after completion of migration. Our results thus demonstrate that γ-crystallins can serve important extralenticular function as well. The observed cell death after P4 extends the previously established vulnerable period between P0 and P4 in second-order auditory neurons to later stages of terminal differentiation.

## Materials and Methods

### Animals

The *Crygn* transgenic mouse (*Crygn*^*fl*^, GenBank accession number JN950846.1) was provided by the International Knockout Mouse Consortium (IKMC project 41021) and carries a floxed exon II *Crygn* allele (Fig 6A). The locus was genotyped with the primers 5´-TTAGCCCTCTCCTGAACACC-3´, 5´-CCTCGTACAGAGTGATCTGG-3´ and 5´-CTACAT AGTTGGCAGTGTTTGG-3´, which amplify in the wild-type (wt) a 524 bp band and in the mutant locus a 207 bp band. Homozygous C*rygn*^*fl/fl*^ mice were crossed with mice containing the locus *Egr2*::*Cre* [20] in the heterozygous state. Mice carrying two floxed *Crygn* alleles and one *Egr2*::*Cre* allele were designated *Crygn*^*Egr2*^. Mice of both sexes were used and littermates served as controls. Control littermates were either *Crygn*^*wt/fl*^ or *Egr2*::*Cre*;*Crygn*^*wt/fl*^, as no differences between these genotypes were observed throughout the study. The day of birth was taken as P0. All protocols were approved by the local animal care and use committee (LAVES, Oldenburg or the Animal Care and Ethics Committee of the regional board of the Federal State Government of Baden-Württemberg, Germany). All experiments were in accordance with the regulations of the German federal law on the care and use of laboratory animals, and followed the guidelines of the EU Directive 2010/63/EU for animal experiments.

To confirm recombination in the auditory hindbrain, adult *Crygn*^*fl/fl*^ transgenic mice with and without *Egr2*::*Cre* were killed by CO_2_ gassing. Brains were removed from the skull and the SOC region was cut out from 100-μm-thick coronal cryosections. Isolated genomic DNA from this tissue was subjected to PCR using the primers 5´-CCATCGCCATCTGCTGCACG-3´ binding at the very 3´end of the *lacZ* gene and 5´-AGGCATTTACAAACATCTCCCCGG-3´ binding downstream to the floxed exon 2 (Fig 6A and 6B). The expected product size after recombination is 604 bp, whereas the unrecombined sequence is 3,411 bp in length and not amplified under the PCR conditions used (30 s extension).

### Molecular cloning of *Cryg flag* fusion constructs

PCR products including flag coding sequences at the 5´-end were generated with the primers 5’-ATTCTAGAATGGACTACAAAGACGATGACGACAAGGGGAAGATCACCTTCTATGAG-3’ and 5’-ATAGAATTCTCAGTAGAAATCCATGACTC-3’ for *Crygd* and 5’- ATTCTAGAATGGACTACAAAGACGATGACGACAAGGCGCAACGCTCGGGAAAGATC-3’ and 5’-ATAGAATTCCGTACGCAGTTGGGACTAG-3’ for *Crygn* using Phusion polymerase (Thermo Fisher), phosphorylated with polynucleotide kinase (Thermo Fisher), and ligated into the *Eco*RV site of pcDNA3.1/Zeo- (Thermo Fisher, Darmstadt, Germany).

### Primary Antibodies

The mouse Crygn peptide CDNFQDQGFMNRVN that was predicted to be antigenic and to discriminate among related proteins was synthesized at Pineda (Berlin, Germany), conjugated to carrier, and used to immunize rabbits. The rabbit anti-Crygd/e was produced by Genescript (Piscataway Township, NJ, USA) by immunization with the peptide RGFQGRHYECSTDHC that is present in both Crygd and Cryg/e. Antibodies were cleaned up by affinity purification. The primary antibody rabbit anti-Cryg was kindly provided by Dr. Samuel Zigler. All three primary antibodies were used in the following dilutions: immunohistochemistry: 1:50 for anti-Crygd/e and Crygn; 1:200 for anti-Cryg, immunocytochemistry: 1:100, and immunoblotting 1:250 for all three antibodies. The primary antibodies guinea pig anti-GlyT2 (Millipore; Darmstadt, Germany) (1:1,000), guinea pig anti-VGluT1 (Millipore) (1:5,000) and the rabbit anti-GlyT2 (1:1,000) have been characterized previously [59,60]. Mouse anti-flag was obtained from Genescript (1:500).

### Immunolabeling

Immunohistochemistry was performed in wt mice, *Crygn* transgenic mice, gerbils and rats of both genders. Three animals were used for each species and age. Animals were deeply anesthetized with chloral hydrate (700 mg per kg body weight i.p.) and perfused transcardially with 0.01 M phosphate-buffered saline (PBS, pH 7.4), followed by 4% PFA (4% paraformaldehyde in 0.1 M phosphate buffer). Brains were removed and stored in fixative overnight at 7°C. After incubation in 30% sucrose/PBS for cryoprotection, 40-μm-thick (P4) or 30-μm-thick (>P4) coronal sections were cut through the hindbrain, collected in 15% sucrose/PBS, thoroughly rinsed in PBS, and blocked for 1 h in blocking solution (2% bovine serum albumine (BSA), 10% goat serum, and 0.3% Triton in TRIS-buffered saline (pH 7.4)). Primary antibodies were added to the blocking solution. Incubation with agitation was carried out at 4°C overnight. After three rinses in PBS, sections were transferred to carrier solution (0.3% Triton, 1% BSA, 1% goat serum) and treated with the secondary antibodies, goat anti-rabbit conjugated to Alexa Fluor 488, goat anti-rabbit conjugated to Alexa Fluor 568, goat anti-guinea pig conjugated to Alexa Fluor 568, or goat anti-guinea pig conjugated to Alexa Fluor 488 (all diluted 1:500, Thermo Fisher). Sections were incubated 1.5 hrs at RT, rinsed again with PBS, mounted on slides, air dried, and coverslips were mounted with Mowiol. Control experiments were performed by omission of secondary antibodies and resulted in the absence of immunosignals. Images were taken with a BZ 8100 E fluorescence microscope (Keyence, Neu-Isenburg, Germany).

For immunocytochemistry, *Crygd* or *Crygn* flag fusion constructs were transiently transfected into HEK293 cells grown on cover slips. After two days of incubation at 37°C and 5% CO_2_ cells were rinsed with PBS, incubated for 10 min with 4% PFA, rinsed three times in PBS, and blocked for 30 min in blocking solution. Primary antibodies were applied in carrier solution and incubated for 1 h at RT. After three rinses in PBS, the secondary antibodies anti-rabbit conjugated to Alexa Fluor 488 and anti-mouse conjugated to Alexa Fluor 568 were added (both diluted 1:500, Molecular Probes, The Netherlands). Cover slips were incubated 1.5 hrs at RT, rinsed again with PBS, mounted on slides with Mowiol and air dried. Images were taken with a BZ 8100 E fluorescence microscope (Keyence).

### Protein isolation and immunoblot

Brains were lysed in a buffer containing 150 mM NaCl, 15 mM Tris, 1% dodecyl-ß-D-maltoside, and 0.4% iodoacetamide. After incubation for 5 min at 25°C, samples were centrifuged for 5 min at 125,000 g. Protein amount of the supernatant was determined using the Bradford assay. 10 μg of each sample were loaded onto a 10% SDS-polyacrylamide gel system. After separation and electrotransfer onto PVDF membranes, membranes were incubated with primary antibodies (dilution 1:250). After incubation for 2 hrs at room temperature, membranes were washed four times with TBS-T (20 mM Tris, 150 mM NaCl, 1% Tween, pH 7.5) and the secondary antibody donkey anti-rabbit IgG-HRP (Santa Cruz Biotechnology, Heidelberg, Germany) was applied for 1 h. After washing, bound antibodies were detected using an enhanced chemiluminescence assay (GE Healthcare, Hamburg, Germany) and a LAS-3000 documentation system (Fujifilm, Düsseldorf, Germany).

### RNA *in situ* hybridization

Two different *Crygn*-derived PCR products were generated. One PCR product (primers 5’- GGAGCATGGTGACTACCCTG-3’ and 5’-CTGGATCTTTATTGCCTCCGTG-3’) amplified sequences from exon 2 to exon 4, the other product (primers 5’- ATCACTCTGTACGAGGGCAAG-3’ and 5’-CATGCCCACAGGCCGACAG-3’) amplified only exon 2. Both products were cloned into pGEMTeasy (Promega). Sequence verified clones were used as template for transcription of RNA *in situ* hybridization probes in the presence of digoxigenin-11-UTP (Roche Applied Science, Mannheim, Germany). 30-μm-thick coronal cryosections were cut in a cryostate (Leica, Wetzlar, Germany) using 4% PFA fixed brains. After proteinase K treatment (10 μg/ml) and deacetylation (12.5 μl acetate anhydride in 5 ml 0.1 M Triethanolamin + 0.9% NaCl), slices were incubated in hybridization buffer (50% v/v formamide, 5x SSC, 20% v/v blocking solution, 0.2% SDS, 1% n-lauroyl sarcosinate) for 1 h, followed by an incubation overnight with the probes (1 μg/ml in hybridization buffer). Both steps were performed at 55°C. After washing for 30 min each at 45°C with 2xSSC, 0.5xSSC and PBS-0.1%-Tween, slices were incubated for 1 h with blocking solution (1% blocking reagent (Roche Applied Science) in maleic acid buffer (pH 7.5) at RT, followed by incubation with anti-digoxigenin antibody conjugated to alkaline phosphatase (Roche Applied Science) (1:1,000 in blocking solution) for 1.5 hrs at RT. Detection occurred with the substrate NBT/BCIP Stock Solution (Roche Applied Science) diluted 1:50 in AP-Buffer (100 mM Tris-HCl, pH 9.5, 150 mM NaCl, 5 mM MgCl_2_) at RT. Sense probes served as negative controls and yielded no signals.

### Anatomical analyses

Nissl staining was performed on consecutive 30-μm-thick coronal cryosections. The volume of auditory nuclei was calculated by multiplying the outlined area with the thickness of each section [38, 61]. Three animals aged P4 or P25 were used from each genotype and the SOC of both sites were analyzed. Analysis was carried out blind to the respective genotype. Sections were then analyzed using ImageJ, and statistical analysis was performed using two-tailed Student's t test after testing for Gaussian distribution of the datasets.

### Auditory Evoked Brainstem Responses

Five *Crygn*^*Egr2*^ mice and four littermate controls (aged 8–10 weeks) were used to test cochlear and brainstem auditory responses. Experiments were performed in a soundproof chamber (IAC) as described [62]. In short, for stimulus generation and recording a multi-function IO-Card (PCI-6052E, National Instruments, USA) was used, housed in an IBM compatible computer. Sound pressure level was controlled with attenuators and amplifiers (Wulf Elektronik, Frankfurt, Germany). Stimuli were delivered open field to the ear by loudspeakers either placed 3 cm lateral to the animal’s pinna or placed as closed field probe for otoacoustic measurements. Sound pressures were calibrated online prior to each measurement.

Animals were anaesthetized by intraperitoneal injection with a mixture of 0.05 mg/kg b.w. fentanyl dihydrogen citrate (Fentanyl Ratiopharm, Ulm, Germany), 5 mg/kg b.w. midazolam (Dormicum Roche Pharma AG, Grenzach-Wyhlen, Germany Germany), 0.5 mg/kg b.w. medetomidin hydrochloride (Sedator Eurovet Animal Health B.V., Aulendorf Germany). To support the heartbeat and to prevent circulation depression, a single dose of 0.2 mg/kg b.w. atropine sulfate (Atropin B.Braun, Melsungen, Germany) was applied within the first 30 min of anaesthesia. Additional doses of anaesthetics were applied in amounts of 1/5 to 1/3 of the initial dose when needed, usually every hour.

Auditory brainstem responses (ABRs) were measured by averaging the evoked electrical response recorded via subcutaneous silver wire electrodes at the ear (active), vertex (reference), and the back (ground) of the animal. Briefly, ABRs were evoked by click (100 μs), noise burst (1 ms static random phase noise) or pure tone stimuli (3 ms duration, 1 ms rise/fall times, frequencies: 2–45.3 kHz, two septs per octave) of gradually increasing sound pressure in 5 dB steps of intensity from 0 to 100 dB SPL at a repetition rate of 60/sec.

Stimuli were presented in open field by a loudspeaker (DT-911, Beyerdynamic, Heilbronn, Germany) and online calibrated with a microphone (B&K 4191, Bruel & Kjaer, Naerum, Denmark) placed near the animals’ ear. The recorded signal was amplified (80 dB), filtered (0.2–5 kHz) and added for alternating phase or polarity to omit the stimulus artefact and cochlear microphonics. Hearing thresholds were determined for click, noise, and each stimulus frequency as the minimal sound pressure evoking a noticeable potential peak in the expected time window of the recorded signal (for details see [61]. From the ABR recordings to increasing stimulus levels, thresholds were defined as the sound pressure level where a stimulus-correlated response was clearly identified by visual inspection of the averaged signal. ABR waveforms were analysed for consecutive amplitude deflections (waves), with each wave consisting of a starting negative (n) peak and the following positive (p) peak. Peak amplitudes of ABR waves I, II, III, and IV were extracted with a customized program based on peak time and amplitude. ABR wave amplitude (μV) growth functions were constructed for individual ears based on the extracted peaks and plotted as I/O function with increasing stimulus level (from −20 to a maximum of 75 dB above threshold).

Cochlear outer hair cell (OHC) function was assessed by the response strength and response threshold from the growth function and the distortion product audiogram of the cubic distortion product otoacoustic emission (DPOAE). The cubic 2×f1−f2 distortion product of the otoacoustic emission (DPOAE) for f2 = 1.24×f1 and L2 = L1−10 dB were recorded in a soundproof chamber (IAC, Niederkrüchten, Germany) as previously described [63]. In short, frequency pairs of tones were between f2 = 4 kHz and f2 = 32 kHz were presented directly into the ear canal by means of a metal coupler connected to two loudspeakers (DT-911, Beyerdynamic). The emission signals were recorded by a microphone (MK 231, Microtech, Gefell, Germany; Preamplifier Brüel & Kjaer 2670, Naerum, Denmark) connected to the coupler. Emission signals were recorded during sound presentation of 260 ms and averaged four times for each sound pressure and frequency presented. For the DP audiogram, the 2f1—f2 distortion product amplitude was measured at constant L2 of 50 dB SPL with f2 varying from 4 to 32 kHz in four steps per octave. The growth function of the 2f1—f2 distortion product amplitude was measured for L1 ranging from 0 to 65 dB SPL with frequencies f2 ranged between 4 and 32 kHz in half octave steps. Threshold was determined as the L1 sound pressure that could generate a 2f1—f2 signal reliably exceeding about 5–10 dB above noise level with noise level typically at -20 dB SPL.

Auditory steady state responses (ASSRs) were used to investigate the temporal capacity of auditory neurons in the auditory pathways. ASSRs are generated by synchronous neuron discharges phase-locked to the modulation frequency of an amplitude or frequency modulated pure tone [64]. ASSRs were measured with amplitude modulated sinusoidal stimuli (carrier frequency 11.3 kHz). The stimuli were presented between -20 and +40 dB around threshold (dB hearing level) in steps of 20 dB. Stimuli were amplitude modulated between 0.8% and 100% and presented for modulation frequencies between 64 Hz and 2,048 Hz in half-octave steps. Unmodulated stimuli (0%) were presented for control. Responses to 1,114 ms long presentations were averaged for 32 repetitions and transformed into frequency energy spectra by fast fourier transformation. From the fast fourier transformation, signal strength at a given modulation frequency was calculated as signal above noise (in nV) within a 16 Hz frequency window. For statistical analysis, data were compared for statistical difference by means of 2-way repeated measures ANOVA (2-way ANOVA) for the factors genotype and stimulus frequency (Hz) or the factors genotype and stimulation level (dB SPL) (Graphpad Prism 6.0, San Diego, USA) and pair-wise Holm-Sidak's multiple comparisons test as posthoc-test (alpha level corrected for repeated measurements). Mean values are quoted ± standard deviation (SD) or standard error of the mean (s.e.m.) unless otherwise stated. *P* < 0.05 was interpreted as statistical significance.

## Abbreviations

ABR: auditory brainstem response
ASS: auditory steady state responses
DPOAE: distortion product otoacoustic emission
LSO: lateral superior olive
MNTB: medial nucleus of the trapezoid body
MSO: medial superior olive
P: postnatal
SOC: superior olivary complex
wt: wild-type

## References

[1] Wistow G, Wyatt K, David L, Gao C, Bateman O, Bernstein S, et al gammaN-crystallin and the evolution of the betagamma-crystallin superfamily in vertebrates. FEBS J. 2005; 272: 2276–2291. 10.1111/j.1742-4658.2005.04655.x 15853812

[2] Graw J. Genetics of crystallins: cataract and beyond. Exp.Eye Res. 2009; 88: 173–189. 10.1016/j.exer.2008.10.011 19007775

[3] Slingsby C, Clout NJ. Structure of the crystallins. Eye. 1999; 13 (Pt 3b): 395–402. 10.1038/eye.1999.113 10627816

[4] Wistow GJ, Piatigorsky J. Lens crystallins: the evolution and expression of proteins for a highly specialized tissue. Annu Rev Biochem. 1988; 57: 479–504. 10.1146/annurev.bi.57.070188.002403 3052280

[5] Mörner CT. Untersuchungen der Proteinsubstanzen in den lichtbrechenden Medien des Auges. Z. Physiol. Chem. 1893; 18: 61–106.

[6] Vendra VPR, Khan I, Chandani S, Muniyandi A, Balasubramanian D. Gamma crystallins of the human eye lens. Biochim Biophys Acta. 2016; 1860: 333–343. 10.1016/j.bbagen.2015.06.007 26116913

[7] Templeton JP, Wang X, Freeman NE, Ma Z, Lu A, Hejtmancik F, et al A crystallin gene network in the mouse retina. Exp Eye Res. 2013; 116: 129–140. 10.1016/j.exer.2013.08.001 23978599 PMC3888992

[8] Templeton JP, Nassr M, Vazquez-Chona F, Freeman-Anderson NE, Orr WE, Williams RW, et al Differential response of C57BL/6J mouse and DBA/2J mouse to optic nerve crush. BMC Neuroscience. 2009; 10: 90 10.1186/1471-2202-10-90 19643015 PMC2727955

[9] Slingsby C, Wistow GJ. Functions of crystallins in and out of lens: roles in elongated and post-mitotic cells. Prog Biophys Mol Biol. 2014; 115: 52–67. 10.1016/j.pbiomolbio.2014.02.006 24582830 PMC4104235

[10] Ehmann H, Hartwich H, Salzig C, Hartmann N, Clément-Ziza M, Ushakov K, et al Time-dependent gene expression analysis of the developing superior olivary complex. J Biol Chem. 2013; 288: 25865–25879. 10.1074/jbc.M113.490508 23893414 PMC3764792

[11] Friauf E. Developmental changes and cellular plasticity in the superior olivary complex In: Parks TN, Rubel EW, Fay RR, Popper AN, editors. Plasticity of the auditory system. New York: Springer; 2004 pp. 49–95.

[12] Nothwang HG, Ebbers L, Schluter T, Willaredt MA. The emerging framework of mammalian auditory hindbrain development. Cell Tissue Res. 2015 10.1007/s00441-014-2110-7 25636588

[13] Kopp-Scheinpflug C, Tozer AJB, Robinson SW, Tempel BL, Hennig MH, Forsythe ID. The sound of silence: ionic mechanisms encoding sound termination. Neuron. 2011; 71: 911–925. 10.1016/j.neuron.2011.06.028 21903083

[14] Caird D, Klinke R. Processing of binaural stimuli by cat superior olivary complex neurons. Exp Brain Res. 1983; 52: 385–399. 6653700 10.1007/BF00238032

[15] Blaesse P, Ehrhardt S, Friauf E, Nothwang HG. Developmental pattern of three vesicular glutamate transporters in the rat superior olivary complex. Cell Tissue Res. 2005; 320: 33–50. 15714284 10.1007/s00441-004-1054-8

[16] Zhou J, Nannapaneni N, Shore S. Vesicular glutamate transporters 1 and 2 are differentially associated with auditory nerve and spinal trigeminal inputs to the cochlear nucleus. J Comp Neurol. 2007; 500: 777–787. 17154258 10.1002/cne.21208

[17] Rosengauer E, Hartwich H, Hartmann AM, Rudnicki A, Satheesh SV, Avraham KB, et al Egr2::Cre mediated conditional ablation of Dicer disrupts histogenesis of mammalian ventral auditory nuclei. PLoS ONE. 2012; 7: e49503 10.1371/journal.pone.0049503 23152916 PMC3495878

[18] Wiechers B, Gestwa G, Mack A, Carroll P, Zenner HP, Knipper M. A changing pattern of brain-derived neurotrophic factor expression correlates with the rearrangement of fibers during cochlear development of rats and mice. J Neurosci. 1999; 19: 3033–3042. 10191320 10.1523/JNEUROSCI.19-08-03033.1999 PMC6782279

[19] Bradley A, Anastassiadis K, Ayadi A, Battey JF, Bell C, Birling M-C, et al The mammalian gene function resource: the International Knockout Mouse Consortium. Mamm Genome. 2012; 23: 580–586. 10.1007/s00335-012-9422-2 22968824 PMC3463800

[20] Voiculescu O, Charnay P, Schneider-Maunoury S. Expression pattern of a Krox-20/Cre knock-in allele in the developing hindbrain, bones, and peripheral nervous system. Genesis. 2000; 26: 123–126. 10686605 10.1002/(sici)1526-968x(200002)26:2<123::aid-gene7>3.0.co;2-o

[21] Ebbers L, Satheesh SV, Janz K, Ruttiger L, Blosa M, Hofmann F, et al L-type calcium channel Cav1.2 is required for maintenance of auditory brainstem nuclei. J Biol Chem. 2015: 23692–23710. 10.1074/jbc.M115.672675 26242732 PMC4583033

[22] Satheesh SV, Kunert K, Ruttiger L, Zuccotti A, Schonig K, Friauf E, et al Retrocochlear function of the peripheral deafness gene Cacna1d. Hum Mol Genet. 2012; 21: 3896–3909. 10.1093/hmg/dds217 22678062

[23] Maricich SM, Xia A, Mathes EL, Wang VY, Oghalai JS, Fritzsch B, et al Atoh1-lineal neurons are required for hearing and for the survival of neurons in the spiral ganglion and brainstem accessory auditory nuclei. J.Neurosci. 2009; 29: 11123–11133. 10.1523/JNEUROSCI.2232-09.2009 19741118 PMC2743121

[24] Cant NB, Benson CG. Parallel auditory pathways: projection patterns of the different neuronal populations in the dorsal and ventral cochlear nuclei. Brain Research Bulletin. 2003; 60: 457–474. 12787867 10.1016/s0361-9230(03)00050-9

[25] Smith PH, Massie A, Joris PX. Acoustic stria: anatomy of physiologically characterized cells and their axonal projection patterns. J Comp Neurol. 2005; 482: 349–371. 10.1002/cne.20407 15669051

[26] Kolkman JA, Stemmer WP. Directed evolution of proteins by exon shuffling. Nat Biotechnol. 2001; 19: 423–428. 10.1038/88084 11329010

[27] Morrow JA, Collie IT, Dunbar DR, Walker GB, Shahid M, Hill DR. Molecular cloning and functional expression of the human glycine transporter GlyT2 and chromosomal localisation of the gene in the human genome. FEBS Lett. 1998; 439: 334–340. 9845349 10.1016/s0014-5793(98)01390-8

[28] Gomeza J, Ohno K, Hulsmann S, Armsen W, Eulenburg V, Richter DW, et al Deletion of the mouse glycine transporter 2 results in a hyperekplexia phenotype and postnatal lethality. Neuron. 2003; 40: 797–806. 14622583 10.1016/s0896-6273(03)00673-1

[29] Zafra F, Aragon C, Olivares L, Danbolt NC, Gimenez C, Storm-Mathisen J. Glycine transporters are differentially expressed among CNS cells. J Neurosci. 1995; 15: 3952–3969. 7751957 10.1523/JNEUROSCI.15-05-03952.1995 PMC6578198

[30] Friauf E, Aragón C, Löhrke S, Westenfelder B, Zafra F. Developmental expression of the glycine transporter GLYT2 in the auditory system of rats suggests involvement in synapse maturation. J Comp Neurol. 1999; 412: 17–37. 10440707

[31] Hoffpauir BK, Kolson DR, Mathers PH, Spirou GA. Maturation of synaptic partners: functional phenotype and synaptic organization tuned in synchrony. J Physiol. 2010; 588: 4365–4385. 10.1113/jphysiol.2010.198564 20855433 PMC3008845

[32] Henry KR. Auditory brainstem volume-conducted responses: origins in the laboratory mouse. J Am Aud.Soc. 1979; 4: 173–178. 511644

[33] Galbraith G, Waschek J, Armstrong B, Edmond J, Lopez I, Liu W, et al Murine auditory brainstem evoked response: putative two-channel differentiation of peripheral and central neural pathways. J Neurosci Methods. 2006; 153: 214–220. 16406043 10.1016/j.jneumeth.2005.10.017

[34] Toyoshima M, Sakurai K, Shimazaki K, Takeda Y, Shimoda Y, Watanabe K. Deficiency of neural recognition molecule NB-2 affects the development of glutamatergic auditory pathways from the ventral cochlear nucleus to the superior olivary complex in mouse. Dev Biol. 2009; 336: 192–200. 10.1016/j.ydbio.2009.09.043 19818338

[35] Schneggenburger R, Forsythe ID. The calyx of Held. Cell Tissue Res. 2006; 326: 311–337. 16896951 10.1007/s00441-006-0272-7

[36] Gersdorff H von, Borst JGG. Short-term plasticity at the calyx of Held. Nat Rev Neurosci. 2002; 3: 53–64. 11823805 10.1038/nrn705

[37] Mostafapour SP, Cochran SL, Del Puerto N M, Rubel EW. Patterns of cell death in mouse anteroventral cochlear nucleus neurons after unilateral cochlea removal. J Comp Neurol. 2000; 426: 561–571. 11027399 10.1002/1096-9861(20001030)426:4<561::aid-cne5>3.0.co;2-g

[38] Hirtz JJ, Boesen M, Braun N, Deitmer JW, Kramer F, Lohr C, et al Cav1.3 calcium channels are required for normal development of the auditory brainstem. J. Neurosci. 2011; 31: 8280–8294. 10.1523/JNEUROSCI.5098-10.2011 21632949 PMC6622878

[39] Lohmann C, Ilic V, Friauf E. Development of a topographically organized auditory network in slice culture is calcium dependent. J.Neurobiol. 1998; 34: 97–112. 9468382 10.1002/(sici)1097-4695(19980205)34:2<97::aid-neu1>3.0.co;2-6

[40] Lohmann C, Friauf E. Distribution of the calcium-binding proteins parvalbumin and calretinin in the auditory brainstem of adult and developing rats. J. Comp. Neurol. 1996; 367: 90–109. 8867285 10.1002/(SICI)1096-9861(19960325)367:1<90::AID-CNE7>3.0.CO;2-E

[41] Friauf E. Distribution of calcium-binding protein Calbindin-D28k in the auditory system of adult and developing rats. J. Comp. Neurol. 1994; 349: 193–211. 7860778 10.1002/cne.903490204

[42] Bagby S, Harvey TS, Eagle SG, Inouye S, Ikura M. NMR-derived three-dimensional solution structure of protein S complexed with calcium. Structure. 1994; 2: 107–122. 8081742 10.1016/s0969-2126(00)00013-7

[43] Clout NJ, Kretschmar M, Jaenicke R, Slingsby C. Crystal structure of the calcium-loaded spherulin 3a dimer sheds light on the evolution of the eye lens betagamma-crystallin domain fold. Structure. 2001; 9: 115–124. 11250196 10.1016/s0969-2126(01)00573-1

[44] Mishra A, Krishnan B, Raman R, Sharma Y. Ca(2+) and betagamma-crystallins: An affair that did not last. Biochim Biophys Acta. 2016; 1860: 299–303. 10.1016/j.bbagen.2015.06.012 26145580

[45] Suman SK, Mishra A, Ravindra D, Yeramala L, Sharma Y. Evolutionary remodeling of betagamma-crystallins for domain stability at cost of Ca2+ binding. J Biol Chem. 2011; 286: 43891–43901. 10.1074/jbc.M111.247890 21949186 PMC3243557

[46] Liedtke T, Schwamborn JC, Schroer U, Thanos S. Elongation of axons during regeneration involves retinal crystallin beta b2 (crybb2). Mol.Cell Proteomics. 2007; 6: 895–907. 17264069 10.1074/mcp.M600245-MCP200

[47] Fischer D, Hauk TG, Muller A, Thanos S. Crystallins of the beta/gamma-superfamily mimic the effects of lens injury and promote axon regeneration. Mol.Cell Neurosci. 2008; 37: 471–479. 10.1016/j.mcn.2007.11.002 18178099

[48] Thanos S, Bohm MRR, Meyer zu Horste M, Prokosch-Willing V, Hennig M, Bauer D, et al Role of crystallins in ocular neuroprotection and axonal regeneration. Prog Retin Eye Res. 2014; 42: 145–161. 10.1016/j.preteyeres.2014.06.004 24998680

[49] Willaredt MA, Schlüter T, Nothwang HG. The gene regulatory networks underlying formation of the auditory hindbrain. Cellular & Molecular Life Sciences. 2015: Epub ahead of print. 10.1007/s00018-014-1759-0 PMC11113740 25332098

[50] Kandler K, Friauf E. Pre- and postnatal development of efferent connections of the cochlear nucleus in the rat. J. Comp. Neurol. 1993; 328: 161–184. 8423239 10.1002/cne.903280202

[51] Grothe B. New roles for synaptic inhibition in sound localization. Nat Rev Neurosci. 2003; 4: 1–11. 10.1038/nrn1136 12838329

[52] Schwartz IR. The superior olivary complex and lateral lemniscal nuclei In: Webster DB, Popper AN, Fay RR, editors. The Mammalian Auditory Pathway: Neuroanatomy. New York: Springer; 1992 pp. 117–167.

[53] Kulesza RJ, Grothe B. Yes, there is a medial nucleus of the trapezoid body in humans. Front Neuroanat. 2015; 9: 35 10.3389/fnana.2015.00035 25873865 PMC4379933

[54] Jalabi W, Kopp-Scheinpflug C, Allen PD, Schiavon E, DiGiacomo RR, Forsythe ID, et al Sound Localization Ability and Glycinergic Innervation of the Superior Olivary Complex Persist after Genetic Deletion of the Medial Nucleus of the Trapezoid Body. J. Neurosci. 2013; 33: 15044–15049. 10.1523/JNEUROSCI.2604-13.2013 24048834 PMC3858601

[55] Takeuchi T, Kudo T, Ogata K, Hamada M, Nakamura M, Kito K, et al Neither MafA/L-Maf nor MafB is essential for lens development in mice. Genes Cells. 2009; 14: 941–947. 10.1111/j.1365-2443.2009.01321.x 19624757

[56] Marrs GS, Morgan WJ, Howell DM, Spirou GA, Mathers PH. Embryonic origins of the mouse superior olivary complex. Dev.Neurobiol. 2013: 384–398. 10.1002/dneu.22069 23303740 PMC4217651

[57] Altieri SC, Jalabi W, Zhao T, Romito-DiGiacomo RR, Maricich SM. En1 directs superior olivary complex neuron positioning, survival, and expression of FoxP1. Dev Biol. 2015; 408: 99–108. 10.1016/j.ydbio.2015.10.008 26542008 PMC4688081

[58] Altieri SC, Zhao T, Jalabi W, Romito-DiGiacomo RR, Maricich SM. En1 is necessary for survival of neurons in the ventral nuclei of the lateral lemniscus. Dev Neurobiol. 2016 10.1002/dneu.22388 PMC4995159 26914477

[59] Dumoulin A, Rostaing P, Bedet C, Levi S, Isambert MF, Henry JP, et al Presence of the vesicular inhibitory amino acid transporter in GABAergic and glycinergic synaptic terminal boutons. J Cell Sci. 1999; 112 (Pt 6): 811–823. 10036231 10.1242/jcs.112.6.811

[60] Poyatos I, Ponce J, Aragon C, Gimenez C, Zafra F. The glycine transporter GLYT2 is a reliable marker for glycine-immunoreactive neurons. Mol.Brain Res. 1997; 49: 63–70. 9387864 10.1016/s0169-328x(97)00124-1

[61] Rüttiger L, Singer W, Panford-Walsh R, Matsumoto M, Lee SC, Zuccotti A, et al The reduced cochlear output and the failure to adapt the central auditory response causes tinnitus in noise exposed rats. PLoS ONE. 2013; 8: e57247 10.1371/journal.pone.0057247 23516401 PMC3596376

[62] Knipper M, Zinn C, Maier H, Praetorius M, Rohbock K, Kopschall I, et al Thyroid hormone deficiency before the onset of hearing causes irreversible damage to peripheral and central auditory systems. J Neurophysiol. 2000; 83: 3101–3112. 10805704 10.1152/jn.2000.83.5.3101

[63] Engel J, Braig C, Ruttiger L, Kuhn S, Zimmermann U, Blin N, et al Two classes of outer hair cells along the tonotopic axis of the cochlea. Neuroscience. 2006; 143: 837–849. 17074442 10.1016/j.neuroscience.2006.08.060

[64] Kuwada S, Batra R, Maher VL. Scalp potentials of normal and hearing-impaired subjects in response to sinusoidally amplitude-modulated tones. Hear Res. 1986; 21: 179–192. 3700256 10.1016/0378-5955(86)90038-9

